# A scalable approach to optimize traffic signal control with federated reinforcement learning

**DOI:** 10.1038/s41598-023-46074-3

**Published:** 2023-11-06

**Authors:** Jingjing Bao, Celimuge Wu, Yangfei Lin, Lei Zhong, Xianfu Chen, Rui Yin

**Affiliations:** 1 Department of Computer and Network Engineering, The University of Electro-Communications, Tokyo, 182-8585 Japan; 2 Connected Advanced Development Division, Toyota Motor Corporation JP, Tokyo, 471-8571 Japan; 3 VTT Technical Research Centre of Finland, 90571 Oulu, Finland; 4 School of Information and Electrical Engineering, Hangzhou City University, Hangzhou, 310015 China

**Keywords:** Computer science, Scientific data

## Abstract

Intelligent Transportation has seen significant advancements with Deep Learning and the Internet of Things, making Traffic Signal Control (TSC) research crucial for reducing congestion, travel time, emissions, and energy consumption. Reinforcement Learning (RL) has emerged as the primary method for TSC, but centralized learning poses communication and computing challenges, while distributed learning struggles to adapt across intersections. This paper presents a novel approach using Federated Learning (FL)-based RL for TSC. FL integrates knowledge from local agents into a global model, overcoming intersection variations with a unified agent state structure. To endow the model with the capacity to globally represent the TSC task while preserving the distinctive feature information inherent to each intersection, a segment of the RL neural network is aggregated to the cloud, and the remaining layers undergo fine-tuning upon convergence of the model training process. Extensive experiments demonstrate reduced queuing and waiting times globally, and the successful scalability of the proposed model is validated on a real-world traffic network in Monaco, showing its potential for new intersections.

## Introduction

The continual growth of the automobile industry and transportation infrastructure undoubtedly improves daily travel convenience. However, traffic congestion remains a persistent issue that poses significant challenges to individuals, economies, and the environment in various countries^1^. For instance, a recent survey by the real-time traffic data provider INRIX revealed alarming figures for London in 2022: a staggering per capita traffic delay time of 151 h, a cost of 869 US dollars per capita in delays, 546 US dollars in fuel expenses, and an alarming frequency of generating 1 metric ton of carbon emissions every 2.53 days^2^. Such congestion is caused, in part, by the growing demand for transportation that surpasses the available road capacity, necessitating improvements through infrastructure expansion or vehicle reduction^3^. Additionally, suboptimal traffic management strategies may lead to underutilization of roads and facilities, further exacerbating chaos and congestion. To address these challenges effectively, innovative and efficient traffic management solutions are crucial.

Traffic management often involves traffic control, and commonly used traffic control strategies include road-based and vehicle-based control strategies^4^. As a widely used road-based control method, Traffic Signal (TS) provides the most basic order and safety guarantee for road traffic. Advancements in technologies like Internet of Vehicles, Cloud Computing, and Deep Learning (DL) open up new possibilities for enhancing traffic conditions through intelligent Traffic Signal Control (TSC)^5^. Despite these opportunities, many cities still rely on traditional TSC schemes, such as fixed-time and heuristic control methods^6^. Fixed-time method uses historical traffic statistics to determine changing sequence of signals and the duration of every signal, while heuristic control methods lay loop sensors and use the real-time data of the road to make signal changing^7^. Although simple to implement, these methods often fail to cope with the complexities of increasing traffic flow and uncertain traffic conditions on a large scale, thereby yielding unsatisfactory results. In addressing intricate traffic scenarios, numerous optimization-based approaches are advanced with the objective of achieving optimal metrics such as travel delay, throughput, and average travel time^8^. Techniques such as Genetic Algorithms and Swarm Optimization are employed to discern the optimal solution for TSC^9,10^. Furthermore, max pressure, as an optimal control idea to maximize global throughput, is embraced by numerous research as a means to enhance traffic signal management^11^. While these methodologies demonstrate commendable outcomes under certain conditions, they often entail substantial computational expenses and extended duration to ascertain the globally optimal solutions. Moreover, their efficacy in managing progressively intricate traffic dynamics remains a challenge.

Reinforcement Learning (RL), a powerful Machine Learning (ML) technique, shows promise in addressing TSC challenges^12^. While distributed RL methods train local decision models for each intersection independently, they lack global optimality and face difficulties in transferring knowledge to other intersections^13^. On the other hand, centralized approaches require data collection and communication infrastructure that may not scale well, especially with a growing number of intersections^14^. As a solution to these challenges, Federated Learning (FL), an integration model of DL, is emerging, enabling the integration of local knowledge without transmitting sensitive data^15^.

Despite numerous studies integrating FL and RL, the existing research is limited to scenarios where all intersections in the traffic network share the same structure during each training instance, i.e., every intersection in the trained traffic network has an identical configuration^16,17^. While these approaches allow for developing a global model, their applicability is restricted to specific types of intersections. Scholars have attempted a unified representation of agent states to address this limitation, specifically exploring intersection-agnostic state representations for heterogeneous intersections^18^. However, on the one hand, the accumulation of too many factors hinders a deep network’s ability to comprehend the environmental state more effectively^7^. On the other hand, challenges persist in integrating models resulting from variations in action spaces. Therefore, while existing efforts enable the integration of knowledge from locally trained models across multiple intersections, they face challenges in extending applicability to intricate intersection structures and fail to generalize effectively to other intersections with significant deviations from the training dataset.

To overcome these challenges primarily due to diverse intersection types, varying lane configurations, and dynamic phase settings that lead to differences in local model inputs and outputs, we propose a novel TSC scheme based on FL and RL to combine models effectively. By training RL models on limited intersection signal agents and integrating the feature networks learned by all agents through FL, we develop a model that can be seamlessly adapted to other intersections with minor parameter adjustments. The key contributions of this paper are as follows:

- We propose an RL method based on FL, effectively integrating the parameters of local RL models into a cloud federated model to solve the TSC problem more efficiently.
- To ensure the applicability of the integrated FL model to diverse intersection structures and phase groups, we introduce a unified state representation and an action selection method that preserves differences. Through partial aggregation and partial fine-tuning, the RL decision model of TS agent is equipped with the global feature extraction layers that contain extensive representation and the local feature extraction layers that learn local specificity.
- The effectiveness and scalability of our proposed method are demonstrated through extensive simulations on the Cologne from Germany and Monaco traffic network.

The organizational structure of this article is as follows. We present an overview of the relevant research in Related work. Problem formulation describes the problem definition and Methods details the proposed methods from three aspects: RL agent and local training, FL aggregation of RL model, and fine-tuning of RL model. Experiments and results presents simulation results that empirically demonstrate the efficacy of the proposed method. Finally, the conclusion and future research direction are discussed in Conclusion.

## Related work

Traditional TSC methods, including fixed-time TSC, actuating TSC, and adaptive TSC, rely on historical data, expert knowledge, and pre-set assumptions. However, their lack of adaptability leads to inefficient signal phases, particularly in high-traffic areas and complex urban environments. Researchers explore search-based methods^19^ and model optimization, such as tree search^20^, genetic algorithms^21^, and swarm optimization^22^, to address these challenges. Prediction-based TSC methods use road state models learned by dynamic Bayesian networks to predict future traffic conditions and adjust signal lights accordingly^23^. Additionally, techniques like Dynamic Programming^24^ and Fuzzy Logic^25^ are applied to TSC, but as urban areas grow more complex, there is a pressing need for advanced and adaptive TSC solutions to meet modern traffic challenges.

ML approaches, including RL and Meta-learning, are introduced to TSC research. RL has been widely used in many fields such as Autopilot^26^, Natural Language Processing^27^ and Robot Control^28^, and has achieved satisfactory results. Meanwhile, RL is adopted to enhance adaptive TSC approaches due to its data-driven nature. RL’s strength lies in its ability to learn decision policies through iterative experimentation, leading to actions based on environmental feedback rather than relying on predefined rules^29–31^. RL effectively addresses the complex sequential decision problem posed by TSC and outperforms conventional methods. Early RL applications in TSC focus on single intersections, optimizing traffic signal strategies through SARSA-based self-learning control strategies^32^. Researchers also explore RL methods based on connectivity trees and phase-sensitive RL approaches to enhance system efficiency and signal light flexibility^33^.

While conventional RL methods can handle simple learning tasks, their scalability and optimality are limited when dealing with complex environments and continuous state and action spaces. The integration of DL improves agents’ ability to perceive complex environmental states and solve intricate problems. The Deep Q-Network (DQN) algorithm is employed to control individual intersections, utilizing discrete high-dimensional state representation methods^34^. Researchers also redefine rewards and incorporate phase gates in RL approaches, making them more applicable in real-world traffic scenarios^35^. Vision-based fully autonomous DRL agents are developed to perceive and respond to complex and dynamic traffic environments using real-time RGB camera stream data from intersections^36^.

As the number of vehicles increases, the successful application of DRL at individual intersections becomes insufficient to meet practical demands. Scholars have started exploring scenarios involving multiple intersections, aiming to extend DRL’s applicability from single intersections to multi-intersection environments. Various approaches, such as multi-agent Advantage Actor-Critic algorithms and multi-layer stacked graph convolutional neural networks, are proposed to stabilize the learning process, enhance observability, and foster collaboration among intersections^37–39^. Despite its advantages, RL faces challenges in solving TSC problems. Centralized learning models can achieve optimal global results but require excessive communication and computational resources^40^. Distributed learning solutions allocate agents to learn local models, hindering generalization across different intersections^18^. This paper seeks to establish a robust model capable of aggregating knowledge from intelligent agents at various intersections with lower communication and computational costs. The model should be adaptable to different intersections with varying structures and traffic conditions.

## Problem formulation

The paper addresses the TSC problem at multiple intersections, where each traffic light in all directions of an intersection is treated as an intelligent agent. The goal is to enable each TS agent within the road network to make real-time decisions and provide the best TS scheme to reduce traffic congestion. There may be variations in the number of intersections, the number of forks (directions) at each intersection (*K*), and the number of lanes at a particular intersection (*M*). The problem is modeled using RL, and the environment in which the agent interacts is crucial for formulating the TSC problem.

**Figure 1:**
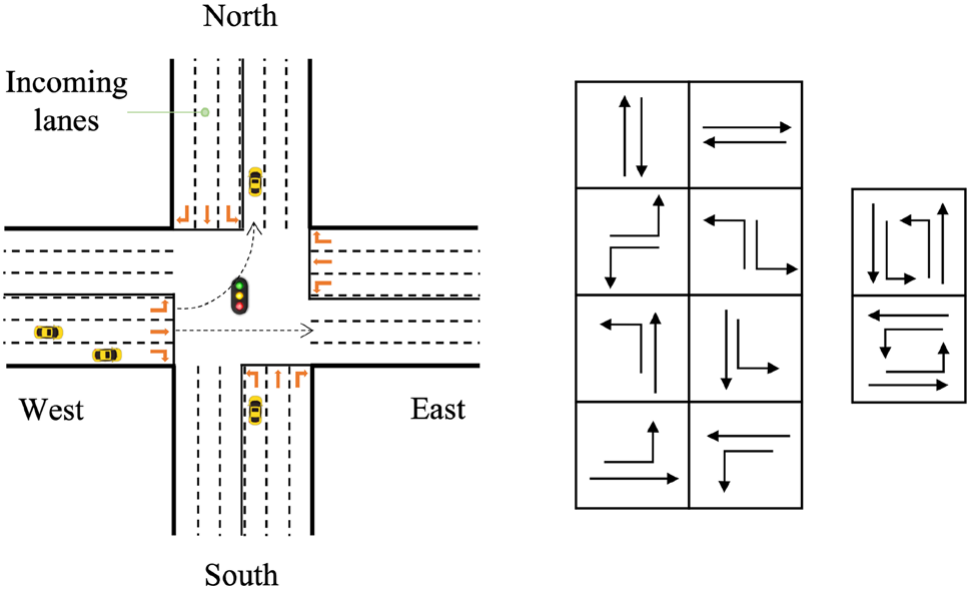
Illustration of intersection and phases.

The environment description includes various aspects. Take Fig. 1 as an example to illustrate below:

- *Incoming lane:* Each intersection has an incoming lane, which is the lane for vehicles entering the intersection. For a specific intersection $g \in G$ (where *G* is the set of all intersections), it has *K* fork roads, and the number of lanes for each fork is $\{M_{1}, M_{2},...,M_{K}\}$. So, the incoming lane of the *k*th fork is recorded as $L=\{l_{1}, l_{2},...,l_{M_{k}}\}$.
- *Movement restriction:* Due to differences in lane settings and traffic flow at each intersection, vehicles allowed to move from the incoming lane to the intersection may vary. For instance, some intersections may not permit left turns in right-hand traffic rules.
- *Phase:* Phases represent the utilization of several lights as a TS at intersections to direct vehicles through the intersection safely. Typically, only non-conflicting driving directions are allowed to pass at any moment. The number of phases may vary depending on the intersection, and certain intersections with low traffic flow may have unique phase settings that allow for simultaneous straight-going and left-turning movements. The right side of Fig. 1 illustrates the possible phases of the intersection shown on the left side.
- *Traffic flow:* Traffic flow varies based on the degree of congestion and may differ at each intersection in terms of time and density. Agents need to make optimal decisions under different traffic flow conditions.

## Methods

**Figure 2:**
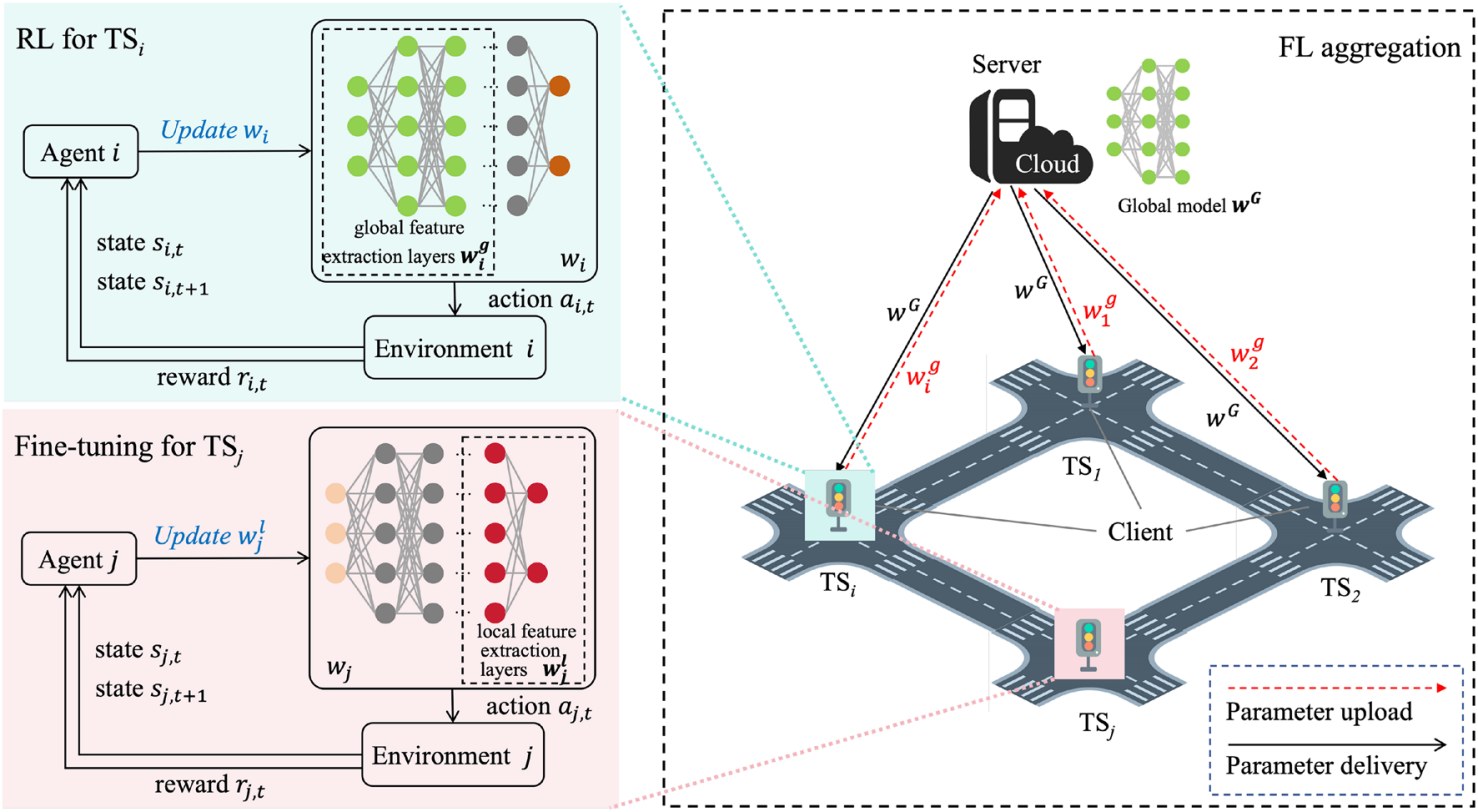
Framework for federated reinforcement learning of TSC.

With reference to the framework in Fig. 2, the proposed method consists of three stages: local training of RL agent, FL aggregation and fine-tuning of the local model. First, RL is used to learn an optimal TSC policy for each intersection with local data. Next, to handle the scalability issue, the approach adopts FL, where RL agents at each intersection act as clients and send partial model updates to the server, and a central server coordinates the training process which aggregates the updates and sends back the updated global model. Finally, after the FL process converges, the remaining parameters are fine-tuned according to the local characteristics of each agent to ensure good performance at new intersections. This approach leverages the collective knowledge of all agents to train more effective global expression and knowledge base applicable to all signals. In parallel, it attempts to provide each agent with a solution to diversity and particularities.

### Local training of reinforcement learning agent

RL is an ML paradigm designed to tackle sequential decision-making problems. At its core is the Markov Decision Process (MDP), which outlines the fundamental elements governing the interaction between an agent and its environment. The TSC problem is a discrete-time RL task, and its MDP can be represented by the tuple $\{S, A, P, R, \gamma \}$. Here, *S* signifies the state space, *A* is the action space, *R* represents the reward generated after the action affects the environment, *P* is the state transition probability, and $\gamma$ is the discount factor that balances immediate reward and future rewards. In the TSC task, the goal of RL is to find an optimal control strategy $\pi ^*$, optimizing the overall performance of vehicles passing through the intersection. The specific agent design is introduced below.

*State representation* To achieve global knowledge integration using FL, a critical aspect is to design a unified representation method for the local RL models learned at intersections of different road structures. For this purpose, an intersection-independent state representation method is established, allowing different action spaces for each agent.

The state representation includes various observation features of the intersection, such as queue length, number of vehicles, position and speed of vehicles, delay time, waiting time, and the current signal phase^41^. This information is collected from various sensors like cameras and radars placed at intersections. The state is represented as a multidimensional matrix:

1 $$\begin{aligned} s = \begin{bmatrix} s_{11}^{1} &{} \cdots &{} s_{1m}^{1} &{} s_{11}^{2} &{} \cdots &{} s_{1m}^{2} &{}\cdots &{} s_{11}^{k} &{} \cdots &{} s_{1m}^{k} \\ s_{21}^{1} &{} \cdots &{} s_{2m}^{1} &{} s_{21}^{2} &{} \cdots &{} s_{2m}^{2} &{}\cdots &{} s_{21}^{k} &{} \cdots &{} s_{2m}^{k} \\ &{} &{} &{} &{} \vdots &{} &{} &{} &{} \\ s_{n1}^{1} &{} \cdots &{} s_{nm}^{1} &{} s_{n1}^{2} &{} \cdots &{} s_{nm}^{2} &{}\cdots &{} s_{n1}^{k} &{} \cdots &{} s_{nm}^{k} \\ \end{bmatrix}, \end{aligned}$$

where $s_{nm}^{k}$ represents the value of the *n*th observation feature of the *m*th incoming lane of the *k*th fork road. In cases where the observed features, number of lanes, or number of forks at the current intersection are smaller than the maximum values, the matrix is filled with 0. This design ensures a uniform representation of the state input while preserving detailed structural information of the intersection, facilitating the model’s ability to learn the mapping from intersection state to TS decisions.

*Action selection* In RL, action refers to the choices and movements made by the agent to interact with the environment. In the TSC problem, actions are typically selected based on two dimensions: time and phrase. Time refers to the decision-making interval, denoted as $\varDelta T$ seconds, allowing the model to flexibly change the phase while ensuring vehicles can safely exit the intersection within one cycle. Phase selection involves each agent choosing one phase from its valid phase set. The number and types of phases vary across intersections based on their road structures and traffic characteristics. If an agent $g \in G$ has *p* phases, its action set is $A = \{a_{1}, a_{2},\ldots ,a_{p}\}$, where each element corresponds to the selection of one phase.

*Reward function* The reward function plays a crucial role in training RL models. The reward in this paper is defined as the weighted sum of the average queue length of halted vehicles (*H*) and the average waiting time of the first vehicle in each lane (*T*) at the intersection. The reward for agent $g \in G$ is:

2 $$\begin{aligned} r = \sum _{l_{m} \in L} (H_{l_{m}} + \sigma T_{l_{m}} ), \end{aligned}$$

where $H_{l_{m}}$ and $T_{l_{m}}$ are the queue length of halting vehicles and waiting time of the first vehicle on $l_{m}$ lane at *g* intersection, respectively. *L* is the incoming lane set of intersection agent *g*. And $\sigma$ is a weight parameter that discounts the waiting time, and its value ranges from 0 to 1. As the vehicles in the experiments have identical properties, the queue length of halted vehicles is replaced by the number of halted vehicles in the queue.

*Reinforcement training algorithm* The DQN^42^ is employed in the proposed method for its simplicity and effectiveness. DQN is a type of model-free RL, and the optimal policy $\pi ^*$is learned through the Q-function. The Q-function is defined as the expected future reward for taking action at in state $s_{t}$, estimated by iterative Bellman update:

3 $$\begin{aligned} Q(s_{t+1}, a_{t+1}) = Q(s_{t}, a_{t}) + \alpha \left( r_{t} + \gamma \max _{a_{t+1}} Q(s_{t+1}, a_{t+1}) - Q(s_{t}, a_{t}) \right) , \end{aligned}$$

where $Q(s_{t}, a_{t})$ is Q-function in time step $t$, $\alpha$ is the learning rate, $\gamma$ is the discount factor, $t$ is the current time step, $t+1$ is the next time step, and $s_{t}$, $a_{t}$ and $r_{t}$ are the state, action and reward of the agent in time step $t$ respectively.

In DQN, a deep neural network is used to approximate the Q-function, denoted as $Q(s, a; w)$, where $w$ are the network weights. To stabilize the learning process, DQN introduces a target network, the weights of which, $w_{t}^-$, are a slowly updated version of the main network weights. This target network is used to compute the target Q-values, in the Bellman update rule. Therefore, the loss function used to train the neural network is:

4 $$\begin{aligned} L(w) = \mathbb {E}_{(s_{t}, a_{t}, r_{t}, s_{t+1}) \sim \text {replay buffer}} \left[ \left( r_{t} + \gamma \max _{a_{t+1}} Q(s_{t+1}, a_{t+1}; w^-) - Q(s_{t}, a_{t}; w) \right) ^2 \right] , \end{aligned}$$

where $w_{t}^-$ is parameter of target network. An experience replay buffer is used to store past transitions (state, action, reward, next state) to break the correlation between consecutive data and improve the stability of learning. During each training iteration, a batch of data is randomly sampled from this buffer for learning.

### Federated learning aggregation

To address scalability issues and avoid transmitting original training data to a centralized server, FL is adopted, which allows model parameters to be transferred instead. FL achieves knowledge integration by aggregating model parameters learned locally on individual data.

Although the FL can improve the generalization ability of the model, various heterogeneous intersections exist in reality. The most obvious intuition is that due to different road structures and requirements, the phase combinations of TS at each intersection are quite different, so it is difficult to unify the output space of the RL model. On the other hand, each area or intersection has certain unique characteristics, which may contain important information about traffic management, which plays an important role in local optimization. These realities put limitations on the model that directly integrates agents at each intersection, and the trained global model may not be able to cope with the situation of new intersections. To this end, the TS agent’s network model is divided into two parts: the global feature extraction layers and the local feature extraction layers. In the federated learning process, only the global feature extraction layers $w^g$ are integrated into the cloud so that it has a generalized representation at the global level, while the local feature extraction layers $w^l$, as a part of the network that retains the unique knowledge of each agent, does not participate in the integration.

As presented in the graphical depiction of Fig. 2, the process involves clients actively sending their global feature extraction layers’ parameters ($w_{i}^g$) to the cloud server after completing a certain number of local RL training, which then conducts aggregation to obtain global feature parameters ($w^G$). Afterward, the cloud server delivers the integrated model parameters to each TS client participating in the training. At this time, each TS agent only needs to update the received model parameters to global feature extraction layers. A one-time federation process is performed at fixed learning intervals (RL training interval) for each round of learning (FL aggregation round). The aggregation algorithm used in this paper is AvgFed^43^, a weighted average fusion method based on the amount of data owned by the local model:

5 $$\begin{aligned} w^G = aggregate( w_{1}^g, w_{2}^g,..., w_{N}^g) = \frac{1}{N} \sum _{i \in N} w_{i}^g, \end{aligned}$$

where *N* is the number of TS agents in the current scenario and $w_{i}^g$ is RL model parameters of global feature extraction layers of TS agent $g_{i}$. $w^G$ is the neural network parameters of the global federated model. Assuming the observation data of each agent in the TSC environment is uniformly distributed, the aggregation is defined accordingly.

### Fine-tuning of the local feature extraction layers

In practical applications of TSC, training a control model for each intersection is unrealistic and not energy-friendly. Therefore, we hope to obtain a model with extensive knowledge and generalization capabilities. To achieve this goal, FL aggregation is first utilized to obtain a global model that learns common features regarding TSC from a wide range of datasets. During the training process, the local feature extraction layers remain local and do not participate in federated aggregation until the model converges. A critical consideration is, when encountering new TSs, local model fine-tuning is performed. This fine-tuning process is to train the neurons of the local feature extraction layers of the new TS agent to adapt to the specific phase settings and other traffic requirements of the TS, thereby achieving better performance in a short time. During this process, the global feature layers remain unchanged, namely, no backpropagation is used to update the global feature layers, which means that the common features learned from the initial broad dataset are retained. During the fine-tuning process, the loss function of TS as an RL agent to update network parameters is as follows:

6 $$\begin{aligned} L(w^l) = \mathbb {E}_{(s_{t}, a_{t}, r_{t}, s_{t+1})} \left[ \left( r_{t} + \gamma \max _{a_{t+1}} Q(s_{t+1}, a_{t+1}; w^{l,-}) - Q(s_{t}, a_{t}; w^l \right) ^2 \right] , \end{aligned}$$

where $w^l$ is the parameters of local feature extraction layers and $w^{l,-}$ is the local feature extraction layers’ parameters of the target network.

This approach aims to enhance the adaptability and efficiency of the system by modifying the new local feature extraction layers to rapidly apply the global model to specific contexts of the new agent. This facilitates the swift adaptation of the global model to specific environments, enabling it to accommodate new TSs with diverse features while incurring low computational costs and training time requirements.

## Experiments and results

### Experimental setting

**Figure 3:**
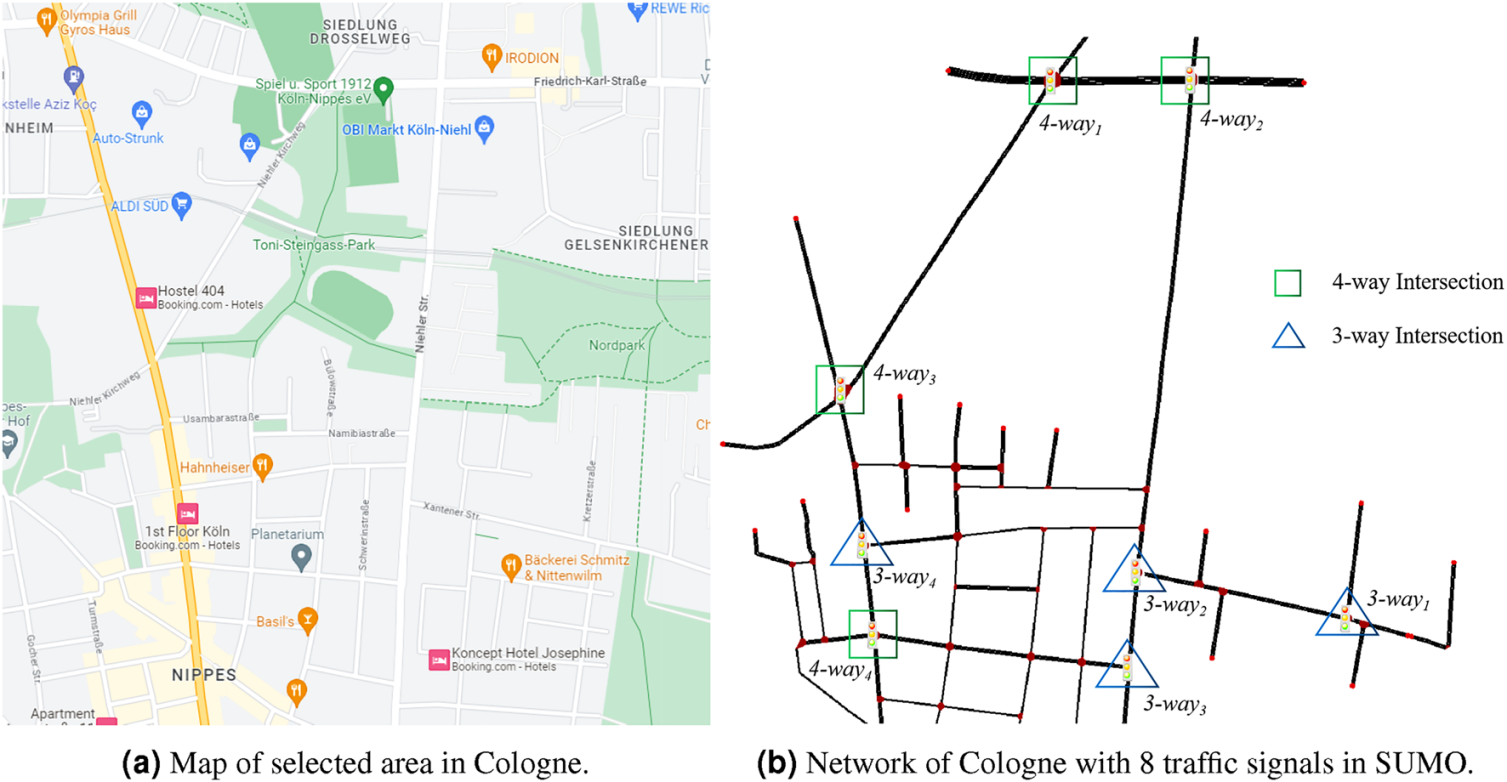
Cologne road network with 8 intersections’ traffic signals.

The experiments are conducted using the SUMO (Simulation of Urban Mobility) simulator, known for its realistic environment and road behavior simulation capabilities. A real road network Fig. 3a in Cologne, Germany, comprising 8 intersections with diverse structures Fig. 3b, is used for the simulations.

The neural network of DQN consists of an input layer, three fully connected feature extraction layers, and an output layer. The numbers of neurons in the hidden layers are 64, 128, and 256, respectively. The output layer parameters are left on the local agent side as the local feature extraction layer and do not participate in aggregation. FL round is set as 20 episodes, where local agents upload their parameters after every 20 training episodes for aggregation and distribution. Other experimental parameters are shown in Table 1.

Four types of models are compared in the experiments:

- *IDQN:* The Independent DQN models without FL are deployed at each intersection, with each agent training its own model independently based on limited local observations.
- *IDQN_tuned:* The IDQN model is fine-tuned to improve its performance in the test, ensuring a fair comparison with the proposed method.
- *Fed_trained:* In the FL process, although the proposed method does not aggregate output layer parameters, each agent holds its own output layer locally. This model serves as a comparison for experiments.
- *Proposed:* The federated global model is deployed at each intersection, and the output layer parameters of each agent are randomly initialized and fine-tuned without the feature layer participating in the fine-tuning.

**Table 1:** Parameters of experiment.

Parameters	Value
Learning rate	0.0001
$\varepsilon$-*greedy* action selection	0.9
Reward discount of RL $\gamma$	0.9
Target network update frequency	30 episodes
Memory capacity	1000
Discount weight of waiting time in reward $\sigma$	0.1

### Results

#### Training process results

**Figure 4:**
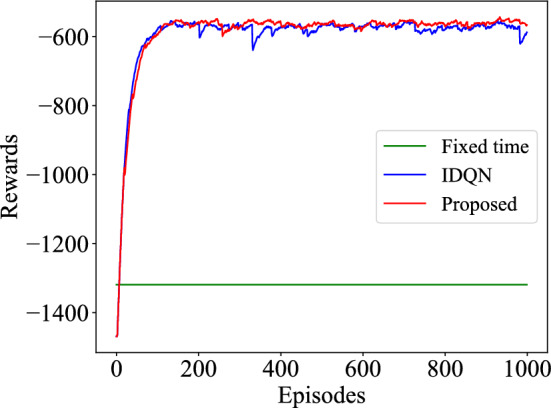
Variation of rewards during training process.

During the training process, the average reward values for each intersection are recorded. As shown in Fig. 4, compared to the fixed-time method with a reward value of $-1319$, the IDQN and the proposed method converged to $-575.17$ and $-562.28$, respectively, indicating the superiority of the DL approach over the fixed-time method. The federated model shows better final convergence, improving by 2.29%. This is likely because the federated model effectively utilizes information from individual intersections, allowing it to adapt better to various traffic conditions and maintain more stable performance.

**Figure 5:**
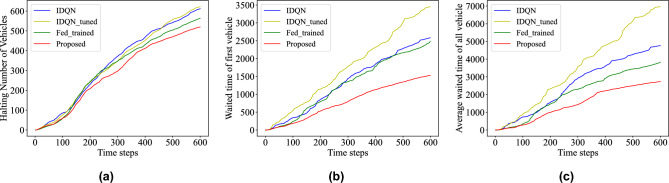
The performance of the proposed method in training data on: (**a**) halting number of vehicles; (**b**) waiting time of first vehicle in every lane; (**c**) average cumulative waiting time of all intersections.

The trained models are tested on the original traffic network using metrics such as the halting number of vehicles at each intersection, the average waiting time of the vehicles at the head of the queue, and the time delay of all vehicles. The results of the four models are plotted in Fig. 5, and the federated model shows significant improvements in all three indicators compared to the independently trained IDQN model. The proposed model also outperforms the Fed_trained model, demonstrating its ability to absorb global knowledge and achieve better results after a few rounds of fine-tuning.

#### Evaluation in different traffic conditions

**Figure 6:**
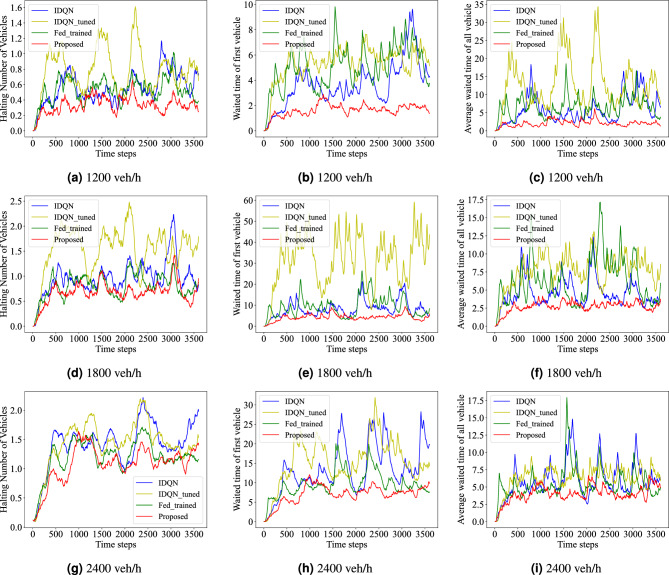
The performance with different density of traffic flow on: halting number of vehicles; waiting time of first vehicle in every lane; average waiting time of all intersections.

To verify the adaptability of the proposed model in different traffic conditions, the density of traffic flow is set as a variable for experiments. The number of vehicles entering the road network per hour is changed to 1200, 1800, and 2400 vehicles per hour. The evaluation indicators include the average halting number of vehicles, the average waiting time of the first vehicle in each lane, and the average cumulative waiting time in all intersections. And the results are the current evaluation value at each time step. The experimental results show that the proposed method consistently outperformed the comparison methods in traffic scenes with different densities. As shown in Fig. 6, when the traffic flow is 1200 veh/h, the proposed method reduces the halting vehicles, the waiting time of the first vehicle, and the waiting time of all vehicles by an average of 39.95%, 55.65%, and 64.48%, respectively, compared to the other models.

**Figure 7:**
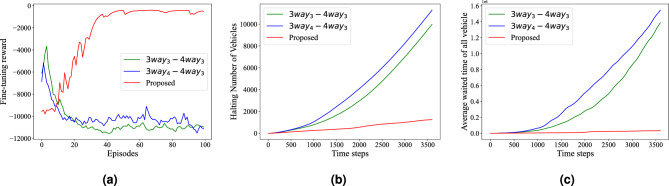
The performance of the proposed method in new road network: (**a**) fine-tuning reward (**b**) halting number of vehicles; (**c**) average cumulative waiting time of all intersections.

#### Experiments of model transplantation

The model trained on 8 TS agents in Cologne is tested for scalability by deploying it on another real-world traffic network in Monaco, which includes 8 four-way intersections and 6 three-way intersections. For the IDQN method, the two groups of models with the best performance are selected for comparison among all combinations and deployed at the intersection of the same structure of the Monaco network. The results are shown in Fig. 7, which demonstrates that the proposed method significantly outperforms the combination of models trained by the IDQN on the Monaco network. As demonstrated in Fig. 7a, even after fine-tuning, the IDQN model fails to achieve similar results. Given that the output layer of the IDQN is an outcome of post-training parameterization, the initial step of fine-tuning yields enhanced performance in comparison to the federated model, which employs random initialization for the output layer. However, despite the initial advantages, the IDQN training ultimately failed due to the fact that only limited features of inputs are learned in the feature layer of the locally trained model and new knowledge cannot be further learned by fine-tuning the output layer independently. On the contrary, the proposed method converged to a better level. In Fig. 7b,c, the testing result of the halting number of vehicles and average waiting time of all vehicles are given. The proposed method maintains fewer vehicles waiting at the intersection, while the comparison method increases significantly. The results indicate that the locally trained IDQN models lack the adaptability required to handle new environments effectively. In contrast, the proposed method, integrated using FL and fine-tuned, demonstrates superior performance when applied to different intersections with varying road structures and traffic conditions.

Overall, the experiments validate the effectiveness and adaptability of the proposed model in various traffic conditions and its ability to outperform locally trained models when applied to new intersections. The fine-tuning process further enhances the model’s ability to adapt to new environments, leading to improved decision-making performance.

## Conclusion

In this paper, we present a novel approach to TSC by integrating RL with FL. The proposed method effectively addresses the scalability challenge of RL by adopting FL, which unifies the state space and integrates feature extraction layer parameters of neural networks. The network of RL model is divided into two parts: the global feature extraction layers and the local feature extraction layers. The former is integrated into a network with global knowledge through FL and sent to the cloud, while the latter learns local unique properties through fine-tuning on the client. Such a learning model better adapts to the output differences and environmental variations among different agents. The evaluation on a real-world traffic network demonstrates the superiority of the proposed approach over the Independent DQN methods in terms of traffic flow efficiency and travel time. Specifically, the convergence performance during training is improved by 2.29%; halting number of vehicles, waiting time of first vehicle in every lane and average cumulative waiting time are improved by an average of 39.95%, 55.65% and 64.48% respectively. In future research, exploring various aggregation algorithms, such as client selection or weight assignment based on performance or reliability, can further optimize model performance. Additionally, incorporating emerging aggregation algorithms based on meta-learning may contribute to further enhancing the performance of FL in tackling TSC problems. This approach represents a promising step towards efficient and adaptive TSC, with potential applications in more complex and diverse urban traffic scenarios, as it has demonstrated superior performance compared to traditional methods.

## Data Availability

The datasets used, generated and analyzed during this study are available from the corresponding author on reasonable request.
